# Development of a novel model for accurate prediction of secondary *Candida* pneumonia in patients with acute exacerbation of chronic obstructive pulmonary disease

**DOI:** 10.3389/fmed.2025.1594934

**Published:** 2025-08-06

**Authors:** Xiaodan Zhu, Changxing Shen

**Affiliations:** ^1^Yiwu Central Hospital, Yiwu, China; ^2^Shanghai Baoshan Luodian Hospital, Shanghai, China

**Keywords:** AECOPD-associated candidiasis, novel model for accurate prediction, infection, cancer, pulmonary function grade

## Abstract

**Background:**

Given the increased risk factors such as the wide application of various dose forms of corticosteroids and broad-spectrum antibiotics in patients with acute exacerbation of chronic obstructive pulmonary disease (AECOPD) in recent years, the incidence of invasive *Candida* pneumonia secondary to AECOPD tends to increase. However, *Candida* infections secondary to AECOPD are often neglected in clinical practice, or even misdiagnosed as bacterial infections, resulting in disease deterioration due to delayed diagnosis. Knowing that early diagnosis and timely treatment can obviously improve the prognosis of pulmonary candidiasis, improving the early diagnosis rate is the key to reduce the mortality of AECOPD-associated candidiasis. The present study was intended to develop a new model that can early and accurately predict the occurrence of *Candida* infections secondary to AECOPD.

**Methods:**

Clinical data of 164 hospitalized patients with AECOPD who received treatment in the department of respiratory medicine of Yiwu Central Hospital between January 2022 and January 2024 were reviewed retrospectively, including the diagnosis, gender, age, BMI, use of inhaled corticosteroids, the duration of using antibiotics, use of carbapenem antibiotics, random blood glucose, albumin level, the presence or absence of cerebral infarction aspiration, cancer chemoradiotherapy, complicated cardiovascular disease, procalcitonin level, pulmonary function grade, and surviving time. Data were treated and analyzed by R language statistical software.

**Results:**

Of the 164 AECOPD patients, 87 were male and 77 were female, with a mean age of 77.28 ± 8.10 years. The model group consisted of 127 AECOPD patients, including 64 with candidiasis secondary to AECOPD and 63 with no candida infection; the validation group consisted of 37 patients, including 14 with secondary candidiasis and 23 with no *Candida* infection. Single factor logistic regression analysis of the patients in the model group showed that BMI, use of antibiotics ≥2 weeks, cancer chemoradiotherapy and pulmonary function grade were four independent predictors for the occurrence of secondary *candida* infection. The weigh factor of the four risk factors was further determined by Multivariate logistic regression analysis as follows: Probability of infection (*P*) = EXP (−17.7063452 + 1.8265388*pulmonary function grade + 1.8443357*cancer chemoradiotherapy + 4. 1749059*use of antibiotics ≥ 2 weeks + 0.4527216*BMI), and *P* > 0.5 suggests the probability of developing secondary candidiasis in the AECOPD patient.

**Conclusion:**

The result demonstrated that this new model could accurately predict the occurrence of secondary candidiasis in AECOPD patients, with an accuracy rate of 84%, thus providing a simple and accurate tool for predicting the probability of secondary candidiasis in AECOPD patients, especially in cancer patients complicated with AECOPD. This model can only be used as an auxiliary assessment tool for the possibility of secondary *candidal* infection and cannot be used as a diagnostic basis.

## Background

Candidiasis is a localized or systemic infectious disease caused by various species of pathogenic *Candida*, which is easy to occur in immunocompromised patients and may invade the local skin, mucosa, tissues and organs of the whole body, with the clinical manifestations and severity of the disease varying individually (1, 2). Given the increased risk factors such as the wide application of various dose forms of corticosteroids and broad-spectrum antibiotics in patients with acute exacerbation of chronic obstructive pulmonary disease (AECOPD) in recent years, the incidence of invasive candidiasis secondary to AECOPD tends to increase. Among various *Candida* infections, candidemia is the most common clinical type of invasive candidiasis, often with poor prognosis or even causing death (3). Candidiasis secondary to AECOPD is often neglected in clinical practice, or misdiagnosed as bacterial infection, resulting in disease deterioration due to delayed diagnosis. Knowing that early diagnosis and timely treatment can obviously improve the prognosis of pulmonary candidiasis, improving the early diagnosis rate is the key to reducing the mortality of *Candida* infections secondary to AECOPD. At present, the diagnosis of secondary candidiasis mainly depends on biophysiological sputum culture and pulmonary CT scan, but the relatively long duration of diagnosis is unfavorable for early diagnosis of the disease; the medical cost of another second-generation sequencing detection technology for sputum specimens is relatively high, and this technology is difficult to popularize; the sensitivity and positivity rate of Candida antigen detection are low (4–7). Knowing that early diagnosis and timely treatment can obviously improve the prognosis of pulmonary candidiasis, improving the early diagnosis rate is the key to reduce the mortality of *Candida* pneumonia secondary to AECOPD. However, there are few studies reporting simple, effective and non-invasive models for predicting the occurrence of AECOPD-associated secondary candidiasis (8). In this study, we report our development of a novel model that can predict the occurrence of secondary candidiasis in AECOPD patients.

## Patients and methods

Included in this study were 164 hospitalized patients with AECOPD who received treatment in the department of respiratory medicine of Yiwu Central Hospital (Yiwu, China) between January 2022 and January 2024. Their clinical data including the diagnosis, gender, age, BMI, use of inhaled corticosteroids (ICS), the duration of using antibiotics, use of carbapenem antibiotics, random blood glucose, albumin level, the presence or absence of cerebral infarction aspiration, cancer chemoradiotherapy, complicated cardiovascular disease, procalcitonin level, pulmonary function grade, and surviving time were collected and analyzed by R language statistical software.

### Inclusion and exclusion criteria

The inclusion criteria were: (1) Pulmonary function meets the diagnostic criteria for COPD (GOLD 2024 version). (2) Chest CT shows typical signs of emphysema. (3) There are complete research related materials in the electronic medical record database. (4) Diagnostic criteria for pulmonary candidiasis: Two or more deep sputum cultures show candidiasis and acute exudation on lung CT. Detailed research methodology explanation: The risk of acute respiratory failure induced by bronchoscopy in AECOPD patients is very high, and none of the patients received bronchoscopy. We obtain deep sputum samples from all patients after breakfast through three steps: rinsing with water– rinsing with compound chlorhexidine mouthwash– rinsing with water– and preparing 5% saline solution for nebulization. This can minimize the interference caused by oral colonization bacteria. Regarding the method of determining acute exudative inflammation of the lungs through chest CT plain scan, the typical CT imaging features of candidal pneumonia are: cloudy or grid like exudative shadows or consolidation shadows in the lung tissue and interstitium around the bronchi, spreading along the bronchi, and significant absorption after antifungal treatment (Follow up chest CT scan after one week of treatment with echinocandin drugs). All of our patients’ CT images can be processed with high-resolution thin-layer imaging through software, and each patient’s CT report is personally reviewed by the associate chief physician to ensure maximum accuracy.

The exclusion criteria were patients: (1) Incomplete case data or unclear diagnosis.

(2) Simultaneously merging other diseases that seriously affect the patient’s survival or easily lead to changes in the condition, such as acute left heart failure, acute ischemic heart disease, rheumatic and immune diseases, severe gastrointestinal bleeding, etc.

### Statistical methods

Grouping based on the diagnosis of secondary pulmonary candidiasis in AECOPD, using R language for data analysis, and comparing the two groups. The above risk factors were analyzed through univariate logistic regression analysis (whether inhaled corticosteroids was used, Use of antibiotics ≥ 2 weeks (Antibacterial drugs that can cover common pathogens of respiratory infections during hospitalization, including carbapenems such as cephalosporins, quinolones, and meropenems. The main focus is on whether the duration of treatment is greater than or equal to 2 weeks. Long course antibiotics can easily cause dysbiosis and secondary *Candida* infections. We will list carbapenems with high antibacterial strength separately, and carbapenems are also very prone to causing bacterial dysbiosis and secondary *Candida* infections), random blood glucose > 11.1 mmol/L, Albumin level < 30 g/L, Post cerebrovascular aspiration, Chemoradiotherapy (Chemoradiotherapy: Patients who have received chemotherapy or radiation therapy within the past 6 months), Complication of cerebrovascular disease, Procalcitonin > 0.5, time of carbapenem antibiotics use ≥ 3 days were defined as binary variables: “Yes” was defined as 1, “No” was defined as 0). The significant results obtained from the one-sided analysis were subjected to multiple logistic regression analysis. *P* < 0.05 is considered statistically significant. The mathematical prediction equation is as follows: m(*P*) = e*^x^*/(1 + e*^x^*), *P* > 0.5 is considered high-risk, while *p* ≤ 0.5 is considered low-risk.

## Results

**FIGURE 1:**
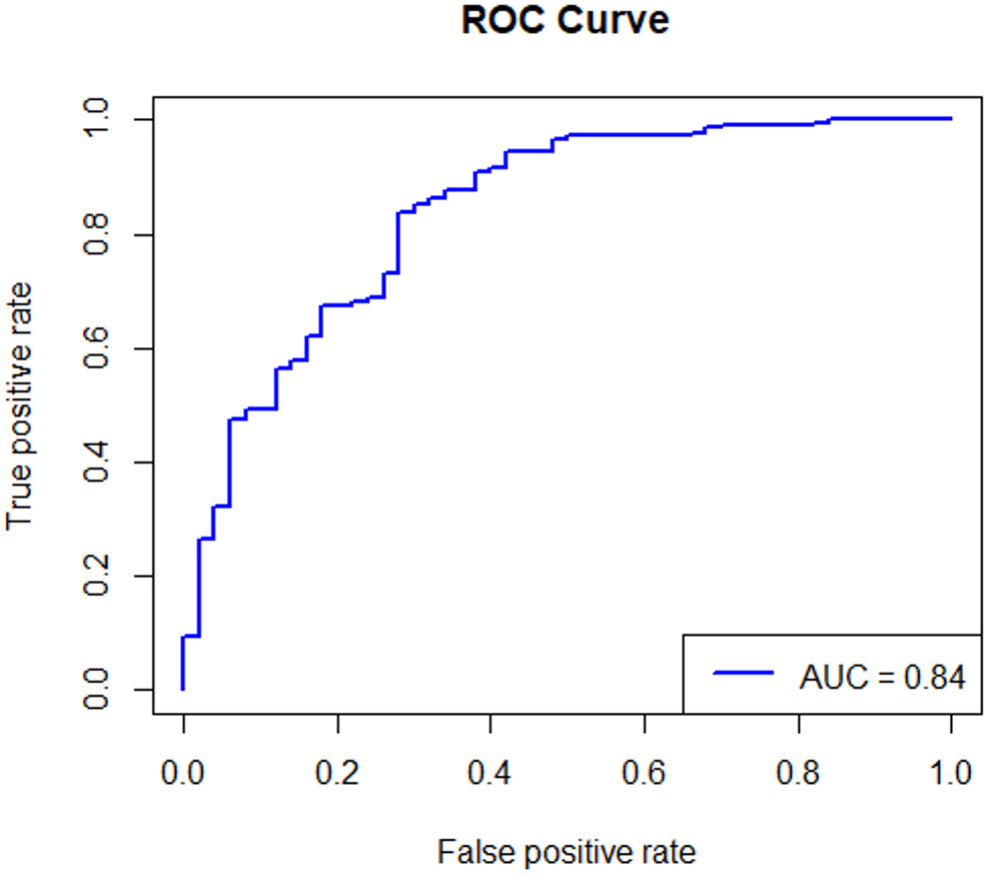
The ROC curve of the predictive performance of the prediction model. Statistical principle: The closer the AUC is to 1.0, the higher the authenticity of the corresponding detection method. The AUC of the prediction model is 0.84, which is close to 1.0, indicating that the prediction model has high application value and high prediction accuracy.

Of the 164 AECOPD patients, 87 were male and 77 were female, ranging in age from 57 to 92 years with a mean of 77.28 ± 8.10 years. All patients have a smoking history of over 20 years. All patients received 3–5 days of methylprednisolone (40 mg iv, once daily) during hospitalization. Of the 127 AECOPD patients in the model group, 64 patients developed secondary *Candida* pneumonia, and the other 63 patients did not. Of the 37 patients in the validation group, 14 patients developed secondary *Candida* pneumonia, and the other 23 patients did not. Univariate logistic regression analysis of the model group showed that BMI, use of antibiotics ≥ 2 weeks, chemoradiotherapy and pulmonary function grade were four independent risk factors for developing secondary candidiasis in AECOPD patients (Table 1). Further multivariate logistic regression analysis was performed to determine the weight factor of the four factors (Table 2), based on which a AECOPD-associated secondary *Candida* pneumonia prediction model in elderly patients was established as follows: Probability of infection (*P*) = EXP (−17.7063452 + 1.8265388*pulmonary function grade + 1.8443357*cancer chemoradiotherapy + 4.1749059*use of antibiotics ≥ 2 weeks + 0.4527216*BMI), and *P* > 0.5 suggests the probability of secondary *Candida* pneumonia occurrence of the COPD patient. In this equation, pulmonary function is expressed as a GOLD grade, where GOLD grade 1 means FEV1%pred ≥ 80% (mild), which is expressed as 1; chemoradiotherapy is expressed as yes = 1 and 0 as no; 3) use of antibiotics is expressed as ≥ 2 weeks = 1 and 0 = no; BMI is calculated as BMI = *body* weight ÷ height2 (Kg/m2). The predictive performance of the prediction model was validated. The result showed that the sensitivity of the prediction model in diagnosing AECOPD-associated secondary *Candida* pneumonia was 0.88, specificity was 0.78, positive prediction value was 0.82, negative prediction value was 0.86, and accuracy was 0.84 (Figure 1 and Table 3).

**TABLE 1:** Univariate logistic regression analysis of independent risk factors for developing *Candida* pneumonia secondary to acute exacerbation of chronic obstructive pulmonary disease (AECOPD).

Factor	b	Z	*P*
Gender	−0.133	−2.567	0.78
Age	0.000561	0.026	0.98
BMI	−0.257	−2.04	0.04*
Use of ICS	19.01	0.014	0.99
Use of antibiotics ≥ 2 weeks	3.35	6.478	0.00*
RBG > 11.1	16.6	0.012	0.99
Albumin < 30 g/L	0.314	0.765	0.44
Post CIA	16.598	0.012	0.99
Chemoradiotherapy	1.507	2.53	0.01*
Complication of CVD	0.087	0.19	0.85
Procalcitonin > 0.5 ng/ml	18.902	0.013	0.99
Use of CPA ≥ 3 days	17.81	0.017	0.98
PF grade	1.7086	5.025	0.00*

*Significant difference. b value, regression coefficient; Z value, b/standard error; BMI, body mass index; ICS, inhaled corticosteroids; RBG, random blood glucose; CIA, cerebrovascular aspiration; CVD, cerebrovascular disease; CPA, carbapenem antibiotics; PF, pulmonary function.

**TABLE 2:** Multivariate logistic regression analysis of independent risk factors for developing *Candida* pneumonia secondary to acute exacerbation of chronic obstructive pulmonary disease (AECOPD).

Factor	b	Z	*P*
BMI	0.4527	1.988	0.04*
Use of antibiotics ≥ 2 weeks	4.1749	5.464	0.00*
Chemoradiotherapy	1.844	2.157	0.03*
PF grade	1.8265	4.043	0.00*

*Significant difference. b value, regression coefficient; Z value, b/standard error; BMI, body mass index; PF, pulmonary function.

**TABLE 3:** Validation data of the predictive performance of the acute exacerbation of chronic obstructive pulmonary disease (AECOPD)-associated secondary *Candida* pneumonia prediction model.

Prediction		Actual value	
		Positive	Negative
Prediction	Positive	77	17
	Negative	10	60

## Discussion

Denning et al. (9) retrieved 85 articles from the literature about disease burden in individual countries and the whole world from 2010 to 2023, and estimated the crude death rate (CDR) and attributable mortality (AM) by comparing differences in the death rate between patients who received treatment and those without receiving treatment and calculating the survival rate of patients who received treatment. After analyzing data from more than 120 countries, they found that each year more than 2,113,000 people contracted invasive pulmonary mycosis from AECOPD, intensive care, lung cancer or hematological malignancies, causing an annual CDR of 1,801,000 people (85.2%); 1,565,000 people contracted *Candida* bloodstream infection or invasive candidiasis, causing 995000 deaths (63.6%). Among them, there may be some AECOPD patients who failed to be diagnosed with complicated candidiasis. The above data indicate that candidiasis secondary to AECOPD seriously affects patient life and health, and therefore early detection and early treatment can undoubtedly reduce the occurrence of invasive candidiasis in AECOPD patients. However, candidiasis often lacks specific symptoms and signs in the early stage and is easy to be misdiagnosed as bacterial infections. In China, the clinical diagnosis of candidiasis mainly depends on a small number of experienced clinicians who proactively assess the risk of *Candida* pneumonia and then undertake further microbial culture to confirm the diagnosis. If a specific model for predicting *Candida* pneumonia in AECOPD patients is available and can be used as a routine in clinical practice, the risk of developing *Candida* pneumonia in AECOPD patients would be reduced substantially by taking anti-fungal therapy in the early stage. Although there is G test [G test, also known as the (1–3) *-*β *- D glucose test*] to assist the diagnosis of *candida* infection at present, it cannot be used by clinicians as a routine practice to assess the risk of *Candida* pneumonia in each patient. In addition, G test may already be lagging behind when the patient presents acute symptoms of fever, chest suffocation and dyspnea, knowing that early intervention is the key to obtaining a relatively good life prognosis. On the other hand, if we can make an extensive and dynamic assessment during the therapeutic process of AECOPD, we are able to identify the risk of *Candida* pneumonia and take early and timely measures to prevent its occurrence or even cure it (10). In AECOPD patients with secondary *Candida* pneumonia, although elderly male smokers are more common, logistic regression analysis suggests that there is no significant relationship between gender and age of patients and secondary *Candida* infections. Other related studies have not found a significant relationship between the risk of secondary *Candida* pneumonia and gender and age (11, 12). The poorer the pulmonary function, the higher the risk of acute exacerbation would be. As a result, the dosage of antibiotics and corticosteroids (either through inhalation, IV injection or oral administration) that the patient requires would be increased, which may further aggravate respiratory microbiota and intestinal microbiota disturbances and compromise their immunity, thus forming a vicious cycle and increasing the mortality of these AECOPD patients (13–15). For AECOPD patients with poor pulmonary function at risk of acute aggravation, inhalation of large doses of ICS is a standard treatment recommended by the GOLD guidelines. However, the risk of localized *Candida* infection progressing to involving the lower respiratory tract is increased in patients with reduced pulmonary function and those who cannot use the inhalation device correctly because of accumulation of large amounts of ICS on the oropharyngeal mucosa due to reduced inspiratory ability or improper use of the device (16). It is apparent that the risk of secondary *Candida* pneumonia is apparently increased in patients with poor respiratory function. There are also other studies reporting that the risk of reaggravation of secondary candidiasis secondary to AECOPD is also increased within 180 days (17).

Cancer patients who receive chemotherapy are often subjected to the toxic effects of chemotherapy drugs, including bone marrow suppression, leukocytopenia, and the reduced immune defense ability against pathogenic infections, all of which may cause pulmonary infections including fungal infections, especially caused by *Candida* and *Aspergillus* (18). Studies have demonstrated that hypoproteinemia, prolonged chemotherapy, AECOPD and basic bronchiectasis are risk factors for nosocomial infection of cancer patients during chemotherapy (*P* < 0.05) (19). In addition, studies on chemotherapy-associated *Candida* pneumonia showed that the incidence of candidiasis was increased in patients receiving radiotherapy, and that positive *Candida* was observed in the saliva specimens of 75% patients with oral squamous cell carcinoma, with *Candida* albicans as the most common yeast. Radiotherapy is also a common treatment for lung squamous cell carcinoma. We also often encountered such patients with pulmonary candidiasis in clinical practice, but the mechanism of radiotherapy causing secondary *candidiasis* in lung cancer patients remains unclear and further study is required. What is clear is that cellular immunity and humoral immunity are decreased in lung cancer patients after radiotherapy, chemotherapy and administration of immunosuppressants (20–22). The lower respiratory tract is susceptible to oral *candidiasis*, causing pulmonary candidiasis. Other studies also observed that *Candida albicans* and *Candida tropicalis* isolated from cancer patients who suffered mouth dryness after radiotherapy presented high a higher biofilm formation ability and bacterial strain metabolic activity as compared those isolated from healthy persons, which may be the pathogenic mechanism underlying *Candida* pneumonia in patients with squamous cancer after radiotherapy (23). These findings suggest that single tumor therapy may increase the risk of *Candida* infection. It is obvious that cancer patients complicated with AECOPD are more likely to be affected by *Candida* pneumonia. Therefore, early identification of high-risk patients and take early preventive measures or even early anti-fungal therapy are critical to deal with the unfavorable effects associated with *Candida* infection (24–27). Based on the numerous findings mentioned above, we have developed a new model for clinical prediction of pulmonary candidiasis in AECOPD patients, believing that it is instructive to clinical diagnosis and treatment. The validation data have confirmed that this prediction model is clinically practicable with a positive prediction rate of 0.82 and an accuracy rate of 0.84. Both the model group and validation group patients followed the same study inclusion criteria, the authors establish this clinical prediction model using model group data, and its predictive value was well demonstrated in the validation group data. It will greatly assist clinical doctors in predicting secondary *Candida* infections in AECOPD. At present, there are no other similar prediction models available for reference or even comparison with this prediction model in terms of prediction accuracy.

### Limitations of this research

Currently, there are very few reported models for predicting *Candida* pneumonia, and there is a lack of comparison with other similar models. This prediction model is only applicable to AECOPD patients and has a relatively limited scope of application. In addition, the sample size of this study is relatively small and needs to be further expanded to verify the accuracy of the prediction model.

## Conclusion

The prediction model of *Candida* pneumonia secondary to AECOPD reported herein presents a high prediction efficiency, offering an accuracy rate of 84%. Clinical doctors should keep in mind these 4 main high-risk predictive factors for AECOPD patients, such as, pulmonary function grade, chemoradiotherapy, prolonged antibiotic use, and higher BMI, especially for cancer patients complicated with AECOPD. But, in clinical practice, AECOPD patients may be infected with both bacteria and fungi simultaneously. Clinical doctors need to continuously perform sputum microbiological culture and chest CT imaging examinations to grasp the dynamic changes in the patient’s condition and adjust anti-infective treatment plans in a timely manner. This model can only be used as an auxiliary assessment tool for the possibility of secondary *Candida* infection and cannot be used as a diagnostic basi.

## Data Availability

The original contributions presented in this study are included in this article/supplementary material, further inquiries can be directed to the Corresponding author.

## References

[1] Tufa T Denning D. The burden of fungal infections in ethiopia. *J Fungi (Basel).* (2019) 5:109. 10.3390/jof5040109 31771096 PMC6958437

[2] Fernandes M Camacho C Gouveia C Chambino B Ribeiro A. Subacute invasive pulmonary aspergillosis (IPA) is a challenging diagnosis. *Cureus.* (2022) 14:e32833. 10.7759/cureus.32833 36570116 PMC9778411

[3] Miller R Harris S Porter R Burnett H. Invasive para-aortic Candida glabrata: a multidisciplinary management challenge. *BMJ Case Rep.* (2021) 14:e240710. 10.1136/bcr-2020-240710 34140325 PMC8212177

[4] Jha B Dey S Tamang M Joshy M Shivananda P Brahmadatan K. Characterization of Candida species isolated from cases of lower respiratory tract infection. *Kathmandu Univ Med J (KUMJ).* (2006) 4:290–4. 18603921

[5] Lu G Wang C Wu C Yan L Tang J. Identification of early biomarkers in a rabbit model of primary Candida pneumonia. *BMC Infect Dis.* (2019) 19:698. 10.1186/s12879-019-4320-9 31387541 PMC6685168

[6] Yang Y Zhu X Sun Y Qian K Liu Z. Comparison of next-generation sequencing with traditional methods for pathogen detection in cases of lower respiratory tract infection at a community hospital in Eastern China. *Medicine.* (2022) 101:e32423. 10.1097/MD.0000000000032423 36595873 PMC9794229

[7] Kelly B Pennington K Limper A. Advances in the diagnosis of fungal pneumonias. *Expert Rev Respir Med.* (2020) 14:703–14. 10.1080/17476348.2020.1753506 32290725 PMC7500531

[8] Han S Meng X. Prediction of risk for secondary lower respiratory tract fungal infection during the acute exacerbation phase of COPD. *J Infect Dev Ctries.* (2023) 17:268–75. 10.3855/jidc.16088 36897910

[9] Denning D. Global incidence and mortality of severe fungal disease. *Lancet Infect Dis.* (2000) 24:e428–38. 10.1016/S1473-3099(23)00692-8 38224705

[10] Silva J Ruiz-Camps I Aguado J. [Invasive fungal infection over the last 30 years]. *Rev Iberoam Micol.* (2021) 38:47–51. 10.1016/j.riam.2021.03.003 34294520

[11] Donohue J Kalberg C Emmett A Merchant K Knobil KA. short-term comparison of fluticasone propionate/salmeterol with ipratropium bromide/albuterol for the treatment of COPD. *Treat Respir Med.* (2004) 3:173–81. 10.2165/00151829-200403030-00005 15219176

[12] Cowie R Boulet L Keith P Scott-Wilson C House K Dorinsky P. Tolerability of a salmeterol xinafoate/fluticasone propionate hydrofluoroalkane metered-dose inhaler in adolescent and adult patients with persistent asthma: a 52-week, open-label, stratified, parallel-group, multicenter study. *Clin Ther.* (2007) 29:1390–402. 10.1016/j.clinthera.2007.07.021 17825690

[13] Khijmatgar S Belur G Venkataram R Karobari M Marya A Shetty V Oral candidal load and oral health status in chronic obstructive pulmonary disease (COPD) patients: a case-cohort study. *Biomed Res Int.* (2021) 2021:5548746. 10.1155/2021/5548746 34545329 PMC8449733

[14] Lu C Mao X. Risk of adverse reactions associated with inhaled corticosteroids for chronic obstructive pulmonary disease: a meta-analysis. *Medicine (Baltimore).* (2024) 103:e36609. 10.1097/MD.0000000000036609 38241558 PMC10798756

[15] Yazici O Cortuk M Casim H Cetinkaya E Mert A Benli A. Candida glabrata pneumonia in a patient with chronic obstructive pulmonary disease. *Case Rep Infect Dis.* (2016) 2016:4737321. 10.1155/2016/4737321 27882253 PMC5110868

[16] Raj R Manu M Prakash P Singhal D Acharya S. The effect of 6 months or longer duration of chronic obstructive respiratory disease medication on the oral health parameters of adults. *Spec Care Dentist.* (2018) 38:133–8. 10.1111/scd.12282 29603344

[17] Zuo Y Wang W Chen Q Liu B Zhang F Jin X Candida in Lower respiratory tract increases the frequency of acute exacerbation of chronic obstructive pulmonary disease: a retrospective case-control study. *Front Cell Infect Microbiol.* (2020) 10:538005. 10.3389/fcimb.2020.538005 33117725 PMC7561360

[18] Kushima N Yanagihara T Ikeda T Chen M Hamada N Fujita M. Candida epiglottitis in a patient undergoing chemotherapy for small cell lung cancer: a case report. *Cureus.* (2024) 16:e72607. 10.7759/cureus.72607 39610630 PMC11604245

[19] Bao Q Zhou H Chen X Yang Q Zhou J. [Characteristics and influencing factors of pathogenic bacteria in lung cancer chemotherapy combined with nosocomial pulmonary infection]. *Zhongguo Fei Ai Za Zhi.* (2019) 22:772–8. 10.3779/j.issn.1009-3419.2019.12.07 31874673 PMC6935041

[20] Ahmed N Oluwole O Mahmoudjafari Z Suleman N McGuirk J. Managing infection complications in the setting of chimeric antigen receptor T cell (CAR-T) therapy. *Clin Hematol Int.* (2024) 6:31–45. 10.46989/001c.115932 38817309 PMC11086990

[21] Liang R Lau G Kwong Y. Chemotherapy and bone marrow transplantation for cancer patients who are also chronic hepatitis B carriers: a review of the problem. *J Clin Oncol.* (1999) 17:394–8. 10.1200/JCO.1999.17.1.394 10458258

[22] Ito K Okamoto M Maruyama F Handa K Yamamoto Y Watanabe M Alteration in antibody-mediated immunity in patients with rituximab-combined chemotherapy and incidence of herpes zoster. *Gan To Kagaku Ryoho.* (2010) 37:99–102. 20087040

[23] Leerahakan P Matangkasombut O Tarapan S Lam-Ubol A. Biofilm formation of Candida isolates from xerostomic post-radiotherapy head and neck cancer patients. *Arch Oral Biol.* (2022) 142:105495. 10.1016/j.archoralbio.2022.105495 35839697

[24] Vázquez-Olvera R Volkow P Velázquez-Acosta C Cornejo-Juárez P. Candida bloodstream infection in patients with cancer: a retrospective analysis of an 11-year period. *Rev Iberoam Micol.* (2023) 40:3–9. 10.1016/j.riam.2022.12.002 36872132

[25] Lin S Chen R Zhu S Wang H Wang L Zou J Candidemia in adults at a tertiary hospital in China: clinical characteristics, species distribution, resistance, and outcomes. *Mycopathologia.* (2018) 183:679–89. 10.1007/s11046-018-0258-5 29572768

[26] Ramirez-Garcia A Rementeria A Aguirre-Urizar J Moragues M Antoran A Pellon A Candida albicans and cancer: can this yeast induce cancer development or progression? *Crit Rev Microbiol.* (2016) 42:181–93. 10.3109/1040841X.2014.913004 24963692

[27] Wang X Zhang W Wu W Wu S Young A Yan Z Is Candida albicans a contributor to cancer? A critical review based on the current evidence. *Microbiol Res.* (2023) 272:127370. 10.1016/j.micres.2023.127370 37028206

